# Lipoxin A_4_ yields an electrophilic 15-oxo metabolite that mediates FPR2 receptor-independent anti-inflammatory signaling

**DOI:** 10.1016/j.jlr.2024.100705

**Published:** 2024-11-19

**Authors:** Adolf Koudelka, Gregory J. Buchan, Veronika Cechova, James P. O’Brien, Emily R. Stevenson, Crystal E. Uvalle, Heng Liu, Steven R. Woodcock, Steven J. Mullett, Cheng Zhang, Bruce A. Freeman, Stacy L. Gelhaus

**Affiliations:** 1 Department of Pharmacology and Chemical Biology, University of Pittsburgh School of Medicine, Pittsburgh, PA, USA; 2 Division of Pulmonary and Critical Care Sleep Medicine, University of Pittsburgh School of Medicine, Pittsburgh, PA, USA; 3 Health Sciences Mass Spectrometry Core, University of Pittsburgh, Pittsburgh, PA, USA

**Keywords:** inflammation resolution, arachidonic acid, lipoxin, electrophile, formyl peptide receptor, lipoxygenase

## Abstract

The enzymatic oxidation of arachidonic acid is proposed to yield trihydroxytetraene species (termed lipoxins) that resolve inflammation via ligand activation of the formyl peptide receptor, FPR2. While cell and murine models activate signaling responses to synthetic lipoxins, primarily lipoxin A_4_ (LXA_4_), there are expanding concerns about the reported biological formation, detection, and signaling mechanisms ascribed to LXA_4_ and related di- and tri-hydroxy ω-6 and ω-3 fatty acids. The generation and signaling actions of LXA_4_ and its primary 15-oxo metabolite were assessed in control, lipopolysaccharide-activated, and arachidonic acid-supplemented RAW264.7 and bone marrow-derived macrophages. Despite the expression of catalytically active enzymes required for LXA_4_ synthesis, both LXA_4_ and its 15-oxo-LXA_4_ metabolite were undetectable in all conditions. Moreover, synthetic LXA_4_ and the membrane-permeable 15-oxo-LXA_4_ methyl ester, which rapidly de-esterified to 15-oxo-LXA_4_, displayed no ligand activity for the putative LXA_4_ receptor FPR2. Alternatively, 15-oxo-LXA_4_, an electrophilic α,β-unsaturated ketone, alkylates nucleophilic amino acids and can modulate redox-sensitive transcriptional regulatory protein and enzyme function. 15-oxo-LXA_4_ activated nuclear factor (erythroid related factor 2)-like 2-regulated expression of anti-inflammatory and repair genes and inhibited NF-κB-regulated pro-inflammatory mediator expression. Synthetic LXA_4_ showed no impact on these macrophage anti-inflammatory and repair responses. In summary, these data show an absence of macrophage LXA_4_ formation and receptor-mediated signaling actions of synthetic LXA_4_. Rather, if present in sufficient concentrations, LXA_4_ and other mono- and poly-hydroxylated unsaturated fatty acids synthesized by macrophages would be readily oxidized to electrophilic α,β-unsaturated ketone products that modulate the redox-sensitive cysteine proteome via G-protein coupled receptor-independent mechanisms.

## Graphical abstract

**Figure:**
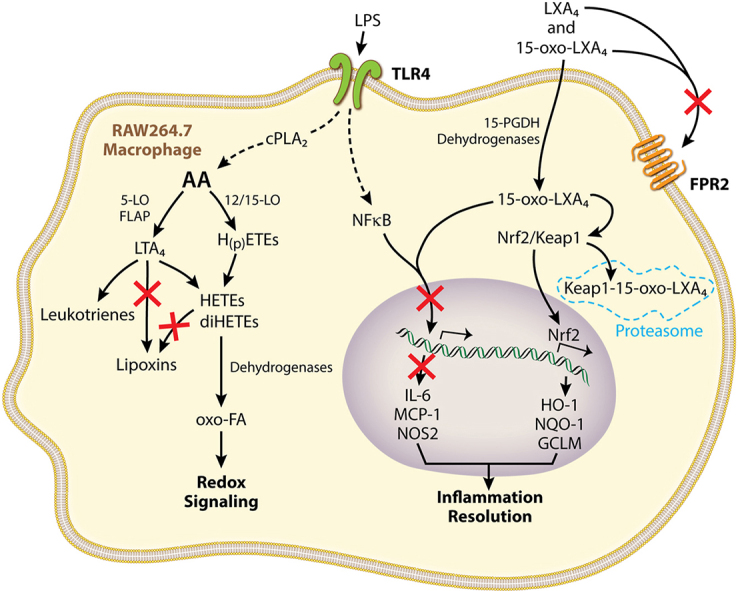

Inflammatory responses initiate diverse free radical and enzymatic oxidation reactions of unsaturated fatty acids, yielding a broad array of products that can orchestrate both pathogenic and tissue-protective responses (1, 2, 3). A subset of unsaturated di- and tri-hydroxy fatty acid lipid mediators, termed lipoxins, resolvins, maresins, protectins, etc., are proposed to be products of the enzymatic oxygenation of arachidonic acid (AA, 20:4), eicosapentaenoic acid (EPA, 20:5) and docosahexaenoic acid (DHA, 22:6) (4, 5). For the AA-derived lipoxins of focus herein [specifically 5*S*,6*R*,15*S*-trihydroxy-7*E*,9*E*,11*Z*,13*E*-eicosatetraenoic acid; lipoxin A_4_, LXA_4_], trihydroxytetraene formation requires multiple oxygenation reactions catalyzed by cells expressing 5-lipoxgenase (LO), 5-LO-activating protein (FLAP), and 12/15-LO. Two major routes of lipoxin synthesis have been proposed. The first putatively occurs in platelets where 5-LO-derived leukotriene A_4_ is a substrate for 12-LO and the second involves the action of two lipoxygenases, 5-LO and 12/15-LO, in leukocytes. Based on previous studies it is most likely that 5-LO and FLAP catalyze the first oxygenation of AA, which is then followed by the second oxygenation by 12/15-LO in the same cell or through transcellular synthesis, requiring intercellular diffusion to a neighboring leukocyte (6, 7, 8, 9).

The inactivation of LXA_4_ bioactivity is proposed to result from the further oxidation of the C15 hydroxyl group by a dehydrogenase such as 15-hydroxy prostaglandin dehydrogenase (15-PGDH), yielding an α,β-unsaturated ketone (10). The broad class of electrophilic α,β-unsaturated ketone metabolites come from diverse sources, including diet, intermediary metabolism, and the autoxidation of unsaturated fatty acids. In aggregate, these metabolites are conferred with an ability to regulate inflammatory and metabolic homeostasis through the post-translational modification of proteins upon Michael's addition of nucleophilic amino acids, primarily cysteine (11). The electrophilic moiety of 15-oxo-5,6-dihydroxy-eicosa-7,9,11,13-tetraenoate (15-oxo-LXA_4_) is inactivated by prostaglandin reductase 2 (PTGR2), yielding the non-electrophilic product 13,14-dihydro-15-oxo-LXA_4_ (Fig. 1) (6, 12, 13, 14).

**Fig. 1:**
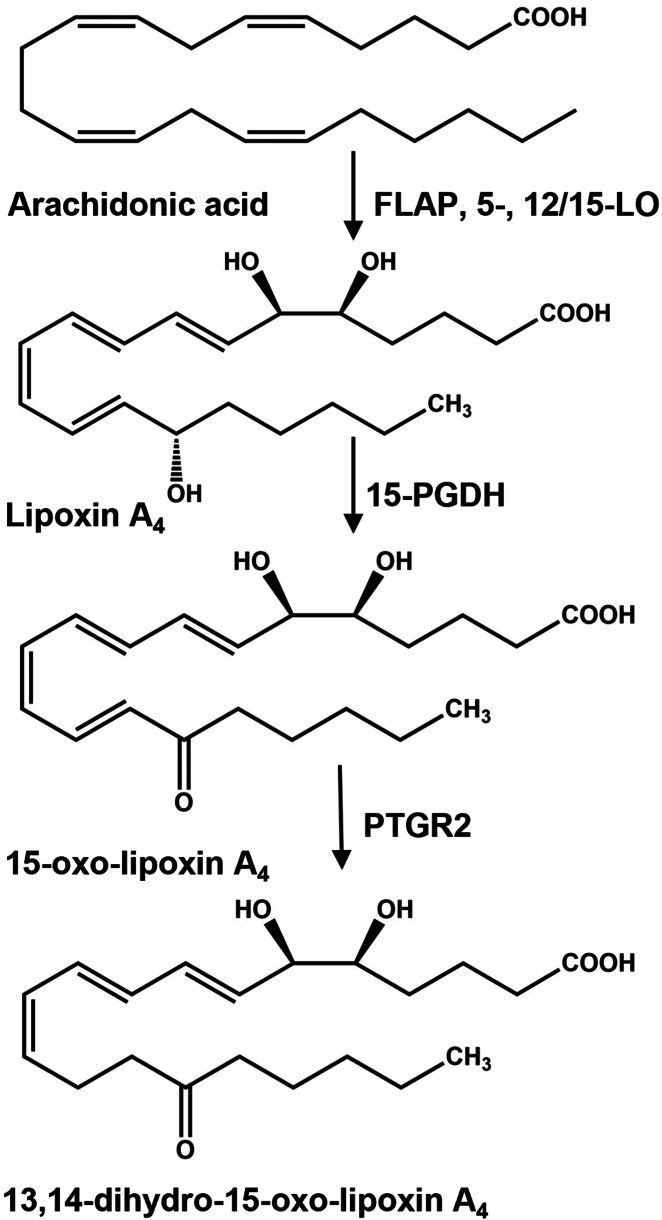
Formation and degradation of lipoxin A_4_. Lipoxin A_4_ (LXA_4_) is produced from the oxygenation of arachidonic acid (AA) by a combination of 5-lipoxygenase (5-LO), 5-lipoxygenase activating protein (FLAP), and 12/15-lipoxygenase (12/15-LO). LXA_4_ is oxidized to an α, β−unsaturated carbonyl-containing fatty acid, 15-oxo-LXA_4_, by 15-hydroxyprostaglandin dehydrogenase (15-PGDH). 15-oxo-LXA_4_ is further metabolized by prostaglandin reductase 2 (PTGR2) via C=C bond reduction at C13-C14 rendering the metabolite 13, 14-dihydro-15-oxo-LXA_4_ non-electrophilic.

Experimental support for the putative anti-inflammatory and tissue repair-related “specialized pro-resolving mediator” (SPM) actions of trihydroxytetraenes stems exclusively from preclinical studies of synthetic LXA_4_ homologs added to biochemical test systems and murine models of inflammatory-related diseases including acute lung injury, asthma, subarachnoid hemorrhage and acute renal failure (8, 15, 16, 17, 18, 19, 20, 21). Prior to the presumed 15-PGDH-mediated inactivation of lipoxins, trihydroxytetraenes are proposed as specific ligands for the formyl peptide receptor (FPR2), the G-protein coupled receptor (GPCR) viewed to transduce LXA_4_ inhibition of inflammatory signaling and promotion of tissue repair (8, 10, 22, 23, 24, 25). Multiple reports have raised concerns about ascribing FPR2 as a transducer of lipoxin signaling, due to contradictory observations coming from (a) FPR2 knockdown and FPR2 deficient model systems, (b) minimal or no changes in GPCR activation-linked cellular Ca^2+^ homeostasis and cyclic AMP levels in test systems treated with synthetic lipoxin homologs, (c) minimal or no β-arrestin recruitment to membranes of LXA_4_-activated cells, (d) the concerning purity of LXA_4_ in commercial preparations, and (e) LXA_4_ instability and undetectability in many biological test systems, all of which are comprehensively reviewed (9, 26).

Recent literature challenges previous data and concepts surrounding the biological generation, endogenous concentrations, and signaling mechanisms of lipoxins and related SPM, initially proposed as endogenous receptor-dependent adaptive signaling mediators. Specifically, multiple labs report undetectable to very low concentrations of SPMs in pre-clinical and human specimens, even after supplementation with low to high concentrations of polyunsaturated fatty acids (27, 28, 29). In the most rigorous determinations, specimens have been analyzed by mass spectrometry-based methods employing synthetic standards, stable isotope-labeled internal standards, and the application of internationally accepted definitions of valid signal-to-noise responses. These analytical approaches are contrasted by less discriminating approaches to SPM bioanalysis where the identification and quantification of many SPM are based on weak or absent peaks in ion chromatograms and mass spectra that are extracted from sample recordings that are not in concordance with synthetic SPM standard product ion spectra (6, 27, 30, 31, 32).

Because endogenously generated lipoxins have not been definitively shown to exert specific signaling responses via FPR2 receptor-dependent mechanisms and do not rise to an in vivo concentration where they might be expected to do so, alternative explanations may account for the anti-inflammatory and adaptive signaling actions of synthetic lipoxin homologs that have been extensively reported in biochemical, cellular, and in vivo test systems (6, 27). Multiple issues form the rationale for the present study, including the (a) short half-life of most polyunsaturated fatty acid signaling mediators (33, 34, 35), (b) very low to no detectable tissue, plasma and urine concentrations of inflammatory-derived trihydroxytetraenes in vivo (6, 27, 36), (c) appreciation that diverse oxidoreductases such as 15-PGDH and dehydrogenase reductase 9 (SDR9C4) rapidly oxidize hydroxyl derivatives of unsaturated fatty acids to electrophilic α,β-unsaturated ketones (10, 25), and (d) knowledge that α,β-unsaturated ketone-containing fatty acid derivatives such as the LXA_4_ metabolite 15-oxo-LXA_4_, are Michael acceptors and can react with redox-sensing nucleophilic centers of small molecules and proteins. These latter reactions can alternatively regulate a broad array of electrophile-sensitive transcriptional regulatory and catalytic protein activities, thus impacting downstream signaling (11, 37, 38, 39).

Herein we report that RAW264.7 macrophages, a cell line commonly used for studies related to the formation of bioactive fatty acids (40, 41), are metabolically competent to mediate LXA_4_ biosynthesis. However, analysis by liquid chromatography-high resolution mass spectrometry (LC-HRMS) revealed that both lipopolysaccharide (LPS)-activated RAW264.7 macrophages and bone marrow-derived macrophages (BMDM) did not generate detectable LXA_4_ and its 15-oxo-LXA_4_ metabolite, even when supplemented with AA. Treatment of RAW264.7 macrophages with 15-oxo-LXA_4_-methyl ester (15-oxo-LXA_4_-Me), a synthetic membrane-permeable precursor for the electrophilic LXA_4_ metabolite 15-oxo-LXA_4_ (42), inhibited NF-ĸB-regulated pro-inflammatory cytokine expression and activated nuclear factor (erythroid related factor 2)-like 2 (Nrf2)-dependent adaptive gene expression responses. In contrast, LXA_4_ had no impact on macrophage Nrf2 target gene expression and NF-ĸB-regulated pro-inflammatory mediator expression. Also, neither LXA_4_ nor 15-oxo-LXA_4_-Me displayed FPR2 ligand activity at low to high concentrations. It was concluded that multi-target electrophile-mediated signaling occurs upon the oxidation of LXA_4_ to 15-oxo-LXA_4_, thus accounting for many of the responses to synthetic LXA_4_ (and related SPMs) when added to ex vivo and in vivo test systems. The present results motivate further investigation into whether signaling-competent trihydroxytetraenes and related SPM occur biologically and if so, whether endogenous concentrations of di- and trihydroxy fatty acids and their electrophilic metabolites are present at levels that would transduce signaling responses via the post-translational modification of redox-sensitive target proteins.

## Materials and methods

### Cell cultivation and treatment

RAW264.7 (ATCC: TIB-71) murine macrophages were grown in DMEM (Corning: 10-013-CV) with 10% FBS (Gibco: 26140-079) and 1% Penicillin/Streptomycin (P/S, Gibco: 15140-122). RAW264.7 cells were plated at 2 × 10^6^ cells/well in 6 well plates and cultured for 12 h before media were replaced with DMEM+1% FBS+1% P/S. Cells were treated with LPS (10 ng/ml, Sigma: L4391, Lot: 067M4036 V), AA (2.5 or 25 μM, Cayman: 90010), LXA_4_ (25 μM, Cayman: 90410), or 15-oxo-LXA_4_-Me (25 μM) which was synthesized as previously (42). Cytotoxicity was determined by MTT [3-(4,5-dimethylthiazol-2-yl)-2,5-diphenyltetrazolium bromide (ThermoFisher: M6494) reduction analysis according to manufacturer’s instructions. Cell lysate and media were collected for analyses including ELISA, Western blot, PCR, and LC-HRMS.

### Bone marrow-derived monocyte/macrophage (BMDM) harvest, differentiation, and treatment

Naïve male C57BL6/J mice (Jackson Laboratories) were housed under standard conditions (12:12 light:dark cycle, 18–24°C) and provided food and water ad libitum. Under a University of Pittsburgh IACUC-approved protocol (#23012457), mice (n = 4) were humanely euthanized via lethal injection of sodium pentobarbital solution (Fatal-Plus, Vortech Pharmaceuticals). The lower limbs of each mouse were thoroughly soaked in 70% ethanol before exposing the hip and knee joints to remove both femurs and tibias as previously described (43). In brief, isolated bones were severed and flushed using ≥ 5 ml ice-cold PBS (2% HI-FBS, Gibco: 10082147) through a 20-gauge needle to collect pooled bone marrow-derived cells. Cells were isolated by centrifugation (400 g, 6 min) and erythrocytes were lysed using ACK lysing buffer (Gibco: A1049201) according to manufacturer instructions. Isolated cells were resuspended in 2% FBS/PBS and passed sequentially through 70 μM and 40 μM sterile cell strainers (Fisher Scientific: 22-363-547/8) for collection. Cells were again pelleted by centrifugation and the concentration of live cells was determined using Trypan Blue solution (0.4%, Gibco, 15250061) exclusion on a hemocytometer. Cells were plated (d0) at 1 × 10^6^ cells/well in 6-well multi-dishes (ThermoFisher Scientific: 150,239) in BMDM media comprised of: 10% FBS, 1% P/S, 2 mM glutathione (ThermoFisher Scientific: 78,259), 20 ng/ml M-CSF (Stem Cell Technologies: 78,059.1) in DMEM (Fisher Scientific: MT10017CV) to initiate monocyte differentiation. Media was exchanged on d3 and d7. Prior to treatment on d8, the media concentration of FBS was reduced to 5%. BMDMs were treated with 10 ng/ml LPS for 6 or 24 h. At the time of collection, media was removed, cells were washed with PBS and detached via incubation with Versene Solution (Gibco: 15040066) for 10 min (37°C). Cells were collected by centrifugation and sedimented cells were washed with cold PBS and again pelleted by centrifugation. Cells were stored at −80°C awaiting downstream preparation and analyses (43).

### Western blot

For Western blot analysis, cells were scraped into ice-cold RIPA buffer (Cell Signaling: 9806) with protease (Roche: 04693132001) and phosphatase (Roche: 4906845001) inhibitors. Samples were further lysed via sonication (10 s on 5 s off, repeat 3x, ThermoFisher: FB120, 4°C) and protein was clarified by centrifugation at 21,000 g for 5 min at 4°C. Protein concentrations were measured using the BCA protein assay (ThermoFisher: 23225) according to the manufacturer’s instructions. Samples were diluted to 2 mg/ml, and mixed with NuPage sample buffer (ThermoFisher: NP0007) and reducing agent (ThermoFisher: NP0009) and heated at 100°C for 10 min. Then, 20 μl of each sample was loaded onto a polyacrylamide gel (ThermoFisher 4%–12%, WG1401BOX and WG1402BOX), 5 μl of dual color standard (Bio-Rad: 1610374) was loaded for molecular weight estimation, and electrophoresis was performed for 1–2 h at 130 V. Protein was transferred to nitrocellulose membrane (Bio-Rad: 1620115) at 100 V for 1 h at 4°C. Membranes were washed with TBS-T buffer and blocked with either 5% milk diluted in TBS-T or 1% casein in TBS (ThermoFisher: 37,532) for 1 h at room temperature. Membranes were washed in TBS-T and primary antibodies FLAP (Invitrogen: PA5-78368), FPR2 (Invitrogen: 720,293), glutamate-cysteine ligase modifier subunit (GCLM Invitrogen: PA5-26111), heme oxygenase 1 (HO-1 Enzo Life Sciences: ADI-SPA-895-F), inducible nitric oxide synthase (iNOS Cell Signaling: 13120), NAD(P)H quinone oxidoreductase 1 (NQO1 Abcam: ab80588), 5-LO (Cell signaling: 3289S), 12/15-LO (Invitrogen: MA5-25891), 15-PGDH (Santa Cruz: SC-271418) were added overnight at 4°C. The following day, primary antibodies were washed with TBS-T, and HRP-linked anti-rabbit (Cell Signaling: 7074) or anti-mouse (Cell Signaling: 7076) secondary antibodies were added. Images were taken using ECL substrates (Bio-Rad: 1705061) and a Bio-Rad ChemiDoc imager. Optical density was evaluated using Image Lab software (Bio-Rad). The optical density of β-actin (Sigma: A4700) was used as endogenous control. Representative images of all western blots are presented for each figure.

### RT-PCR

For PCR analysis, cells were scraped into TRIzol (Invitrogen: 15596026). RNA was isolated, concentration measured, and cDNA prepared as described previously (44). FAM-dyed primers (all purchased from Taqman) were used: *Gclm* (Mm01324400_m1), *Ho1* (Mm00516005_m1), interleukins *Il1b* (Mm00434228_m1) and *Il6* (Mm99999064_m1), monocyte chemoattractant protein 1 (*Ccl2*Mm00441242_m1), *Nos2* (Mm00440502_m1) and *Nqo1* (Mm01253561_m1), with VIC-dyed *Actb* (Mm00607939_s1) used as endogenous control.

### ELISA

Media were centrifuged at 500 *g* for 5 min at 4°C and diluted so that absorbance results were in the range of the standard curve. Specific protocols for each cytokine came from the Invitrogen kits: IL-1β (88-7013-88), IL-6 (88-7064-88), and MCP-1 (88-7391-88).

### LC-MS sample preparation

Media were collected and cells were washed 3x with PBS, cells were scraped into PBS and immediately frozen at −80°C. LXA_4_-d_5_ (10 μl of 1 μg/ml stock, Cayman: 10007737) was added to 1 ml of cell lysate or media. To each sample, 4 ml of chloroform:methanol (2:1) was added, vortexed, and centrifuged at 2500 rpm for 10 min at 4°C. The organic layer was dried under nitrogen and solvated in 100 μl methanol on the day of analysis.

### LC-HRMS

Samples were analyzed on a Thermo Fisher Exploris 240 hybrid mass spectrometer coupled to a Vanquish Horizon UHPLC. Samples were applied to a Luna C8 column (2 × 100 mm, Phenomenex: 00D-4248-B0) and eluted with a linear gradient using H_2_O with 0.1% acetic acid as solvent A and acetonitrile with 0.1% acetic acid as solvent B. Samples were loaded at 35% B and the gradient increased to 60% B over 30 min, held at 100% B for 2 min, and equilibrated at 35% B for 3 min at a flow rate of 0.3 ml/min. LXA_4_ and LXA_4_ metabolites were measured using negative electrospray ionization under the following MS conditions: source, 2600 V; sheath gas 50, auxiliary gas 10, sweep gas 1, ion transfer temperature 325°C, and vaporizer temperature 350°C. A collision energy of 35 was used for data dependent MS^2^ at a resolution of 60K along with a targeted inclusion list of oxylipin accurate masses. Relative levels of LXA_4_ and 15-oxo-LXA_4_ were quantified by normalizing analyte peak area to the peak area of LXA_4_-d_5_.

### 15-Oxo-LXA_4_ GSH adduct standard and LC-MS analysis

A glutathionylated 15-oxo-LXA_4_ standard was made by incubation of 10 μM 15-oxo-LXA_4_ with 100 μM GSH in 50 mM potassium phosphate buffer (pH = 8) for 1 h at 37°C (45, 46). LXA_4_ was oxidized with recombinant 15-PGDH to make 15-oxo-LXA_4_ per the manufacturer’s instructions (R&D systems: 5660-DH). GSH conjugates from the treatment of RAW264.7 macrophages with 25 μM LXA_4_ were extracted from 1 ml of cell supernatant using Oasis HLB 1 cc solid phase extraction columns (Waters) and applied to a Luna C18 column (2 × 100 mm, Phenomenex) at a flow rate of 0.25 ml/min and eluted with a linear gradient consisting of solvent A (H2O + 0.1% acetic acid) and Solvent B (acetonitrile + 0.1% acetic acid). The gradient started at 20% B at 5 min and increased to 98% B at 25 min. The gradient was held at 100% B for 2 min and equilibrated at 20% B for 35 min. GSH adducts were analyzed on a 6500+ QTRAP coupled to an Exion LC (Sciex) using multiple reaction monitoring (658 → 308) and positive ionization with the following MS conditions: CUR 40, CAD med, IS 4500, GS1 70, GS2 65, Temp 550°C, DP 80, EP 7, CE 17, CXP 7.

### GTPγS binding assay

The GTPγS binding assay was conducted using cell membranes overexpressing FPR2 and purified G_i_ heterotrimer, following our previously reported method (47). Briefly, Sf9 insect cells (ExpressionSystems) were infected with baculovirus expressing FPR2. The cells were collected by centrifugation at 8000 *g* for 10 min after 48 h. For ^35^S-GTPγS binding analysis, ∼200 μg/ml of human FPR2 cell membrane was incubated with 200 nM purified G_i_ protein for 20 min on ice in buffer containing 20 mM HEPES, pH 7.5, 150 mM NaCl, 5 mM MgCl_2_, 3 μg/ml BSA, 0.1 μM TCEP, and 5 μM GDP. Next, 25 μl FPR2-G_i_ mix was transferred to 225 μl reaction buffer containing 20 mM HEPES, pH 7.5, 150 mM NaCl, 5 mM MgCl_2_, 3 μg/ml BSA, 0.1 μM TCEP, 1 μM GDP, 35 pM ^35^S-GTPγS (PerkinElmer) and ligands (LXA_4_ and 15-oxo-LXA_4_-Me at 2 μM, WKYMVm (Tocris) at 5 μM). After an additional 15 min incubation at 25°C, the reaction was terminated by adding 6 ml of cold wash buffer containing 20 mM HEPES, pH 7.5, 150 mM NaCl, and 5 mM MgCl_2_, and filtering through glass fiber prefilters (Millipore Sigma,). After washing 4 times with 6 ml cold buffer, the filters were incubated with 5 ml of CytoScint liquid scintillation cocktail (MP Biomedicals) and counted on a Beckman LS6500 scintillation counter.

### Statistical analysis

was performed using GraphPad Prism 9 (GraphPad Software). Bar graph data report mean values of all results ± SD, with each individual result represented by a black dot. All data presented in the bar graphs were tested via a 2-way analysis of variance. Symbols in XY graphs represent mean values of all results ± SD. Data were tested via nonlinear regression (curve fit), represented by a connecting curve. Data represents 3–4 independent experiments. Statistically significant results are denoted by asterisks, with ∗ *P* ≤ 0.05, ∗∗*P* ≤ 0.01, ∗∗∗*P* ≤ 0.001, ∗∗∗∗*P* ≤ 0.0001.

## Results

### RAW264.7 macrophages express active enzymes required for the oxidation of AA to LXA_4_

**Fig. 2:**
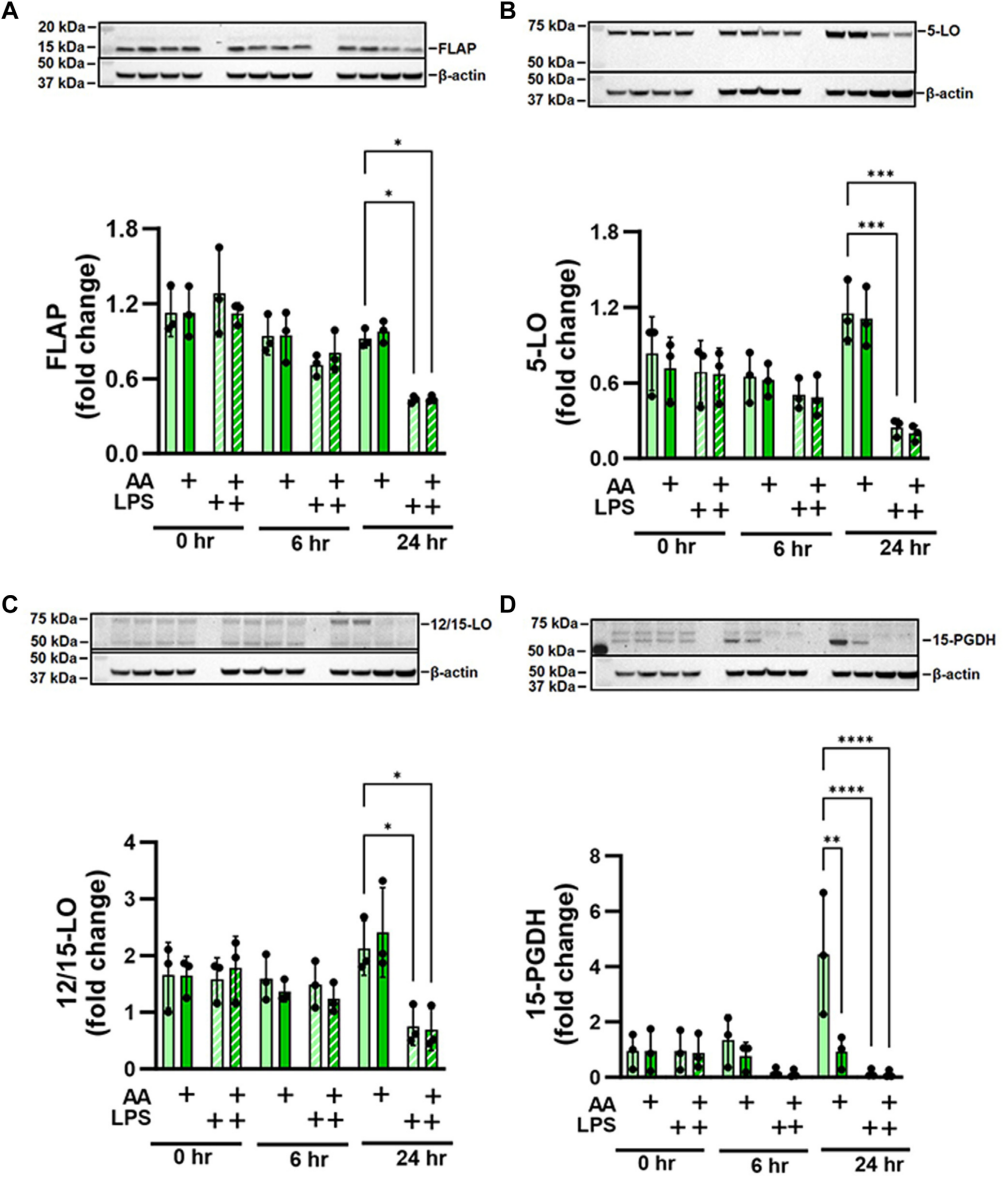
Protein expression of lipoxin biosynthetic enzymes. RAW264.7 macrophages express (A) 5-lipoxygenase activating protein (FLAP), (B) 5-lipoxygense (5-LO), (C) 12/15-lipoxygenase (12/15-LO), and (D) 15-hydroxyprostaglandin dehydrogenase (15-PGDH) at basal conditions and upon stimulation with LPS and/or arachidonic acid supplementation over a 24 h time course. Immunohistochemical results are representative of three independent experiments, ∗*P* < 0.05, ∗∗*P* < 0.01, ∗∗∗*P* < 0.001, and ∗∗∗∗*P* < 0.0001.

The formation of the AA hydroperoxide regioisomers that are the precursors for the endogenous synthesis of LXA_4_ is catalyzed by consecutive oxygenation reactions by FLAP/5-LO, and 12/15-LO (6, 12, 13, 14). All proteins were detectable in RAW264.7 cell lysates over the 24 h study period (Fig. 2A–C). 15-PGDH, which further oxidizes LXA_4_ to 15-oxo-LXA_4_ (48, 49), was expressed throughout the 24 h study period (Fig. 2D). LPS treatment of RAW264.7 macrophages showed modest downregulation of the protein expression of FLAP, 5-LO, 12/15-LO and 15-PGDH (Fig. 2A–D). For subsequent studies, an LPS concentration was selected so that there was a maximum of 20% loss of RAW264.7 cell viability over 24 h, an effect that was mitigated by AA supplementation (supplemental Fig. S1). Under these conditions of LPS activation and AA supplementation (2.5 and 25 μM), hydroxy eicosatetraenoic acids (HETEs) were formed and detected by LC-HRMS at 6 and 24 h (supplemental Fig. S2). Based on the retention times of the internal standards, 15-HETE-d_8_ (21.84 min), 12-HETE-d_8_ (23.27 min), and 5-HETE-d_8_ (24.07 min), the main products formed in the RAW264.7 macrophage were 15-HETE and 11-HETE although a deuterated internal standard was not included for this regioisomer. At the 24 h time point for both concentrations of AA (2.5 and 25 μM), there is a small peak for 5-HETE (supplemental Fig. S2).

### LXA_4_ production was not detectable in macrophages under basal, AA-supplemented, and LPS-activated conditions

**Fig. 3:**
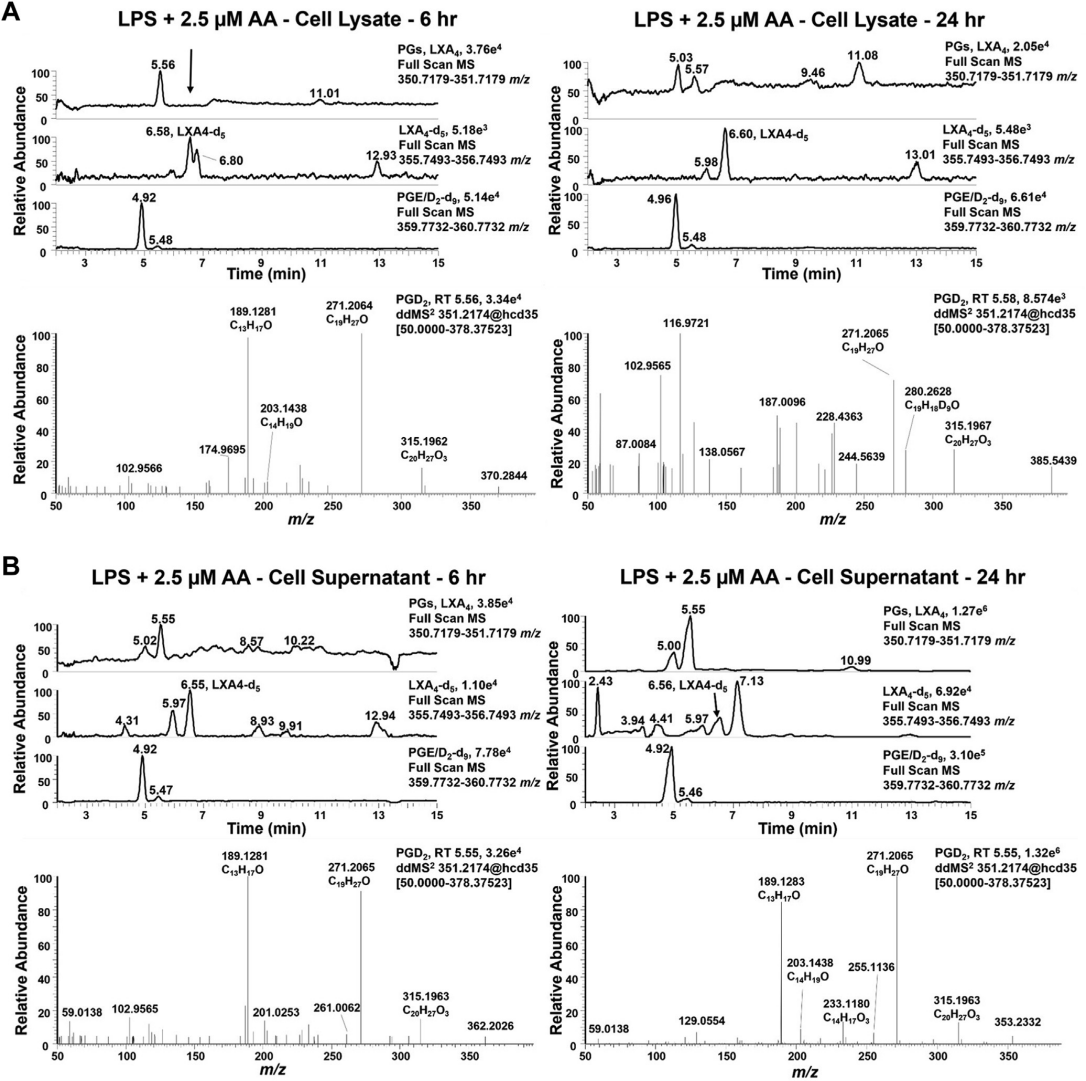
RAW264.7 macrophages do not produce LXA_4_. RAW264.7 macrophages were simultaneously activated with 10 ng/ml LPS and supplemented with 2.5 μM arachidonic acid for 6 and 24 h. A: Cell lysate and (B) cell supernatants were extracted using a modified Folch extraction containing the internal standards LXA_4_-d_5_, PGE_2_-d_9_, PGD_2_-d_9_, 15-HETE-d_8_, 12-HETE-d_8_, and 5-HETE-d_8_ for analysis of oxylipins by LC-HRMS. LXA_4_ was not observed in both cell lysates and supernatants. In cell supernatants oxylipins with the same accurate *m/z* of 351.2183, but different retention times (5.02 and 5.55 min vs. 6.55 min) were observed. These peaks aligned with prostaglandin E_2_ and D_2_ deuterated internal standards with *m/z* 360.2732 and retention times of 4.92 min and 5.47 min, respectively. The MS^2^ for the metabolite at 5.55 min contained diagnostic fragment ions at *m/z* 315.1963, 271.2065, and 189.1281 further confirming a prostaglandin derivative. The extraction ion chromatograms and corresponding spectra are representative of three independent experiments.

Although all enzymes necessary to produce LXA_4_ were present in RAW264.7 macrophage, LC-HRMS analysis did not show a peak with the matching retention time, accurate mass and MS^2^ fragmentation pattern in cell lysates or supernatants after LPS activation (supplemental Fig. S3A) that compared to those of both the LXA_4_ standard (*m/z* 351.2179, M-H+) and the LXA_4_-d_5_ internal standard (*m/z* 355.2494, M-H^+^), both eluting at a retention time of 6.5 min (supplemental Fig. S3B). Even the supplementation of 2.5 μM AA for 6 and 24 h with LPS activation (Fig. 3) or with 25 μM AA at the same time points (supplemental Fig. S4) did not result in detectable LXA_4_. In the cell supernatant and media, two primary peaks with the same accurate mass of LXA_4_ were observed, but at earlier retention times (4.96 and 5.53 min) than both the LXA_4_ standard and the LXA_4_-d_5_ internal standard (supplemental Fig. S3B). Upon further investigation of the MS^2^ and the addition of deuterated internal standards to confirm retention times, the isobaric species at 4.96 and 5.53 min are likely prostaglandin (PG)E_2_ and PGD_2_, as evidenced by the characteristic fragment ions of *m/z* 315.1963, 271.2068, and 189.1281 (Fig. 3 and supplemental Fig. S4). To investigate the formation of LXA_4_ in primary cells, we utilized BMDM from naïve C57BL6/J mice that were cultured and activated with LPS at 6 and 24 h. Like RAW264.7 macrophages, there was no peak detected for LXA_4_ at the retention time observed for the internal standard at either time point in the cell lysate (supplemental Fig. S5A) or supernatant (supplemental Fig. S5B). Prostaglandins were observed in cell lysate at 6 and 24 h after LPS activation as well as the formation of HETEs as shown at 6 h (supplemental Fig. S5C).

### RAW264.7 macrophages oxidize exogenously added LXA_4_ to the electrophilic metabolite 15-oxo-LXA_4_

**Fig. 4:**
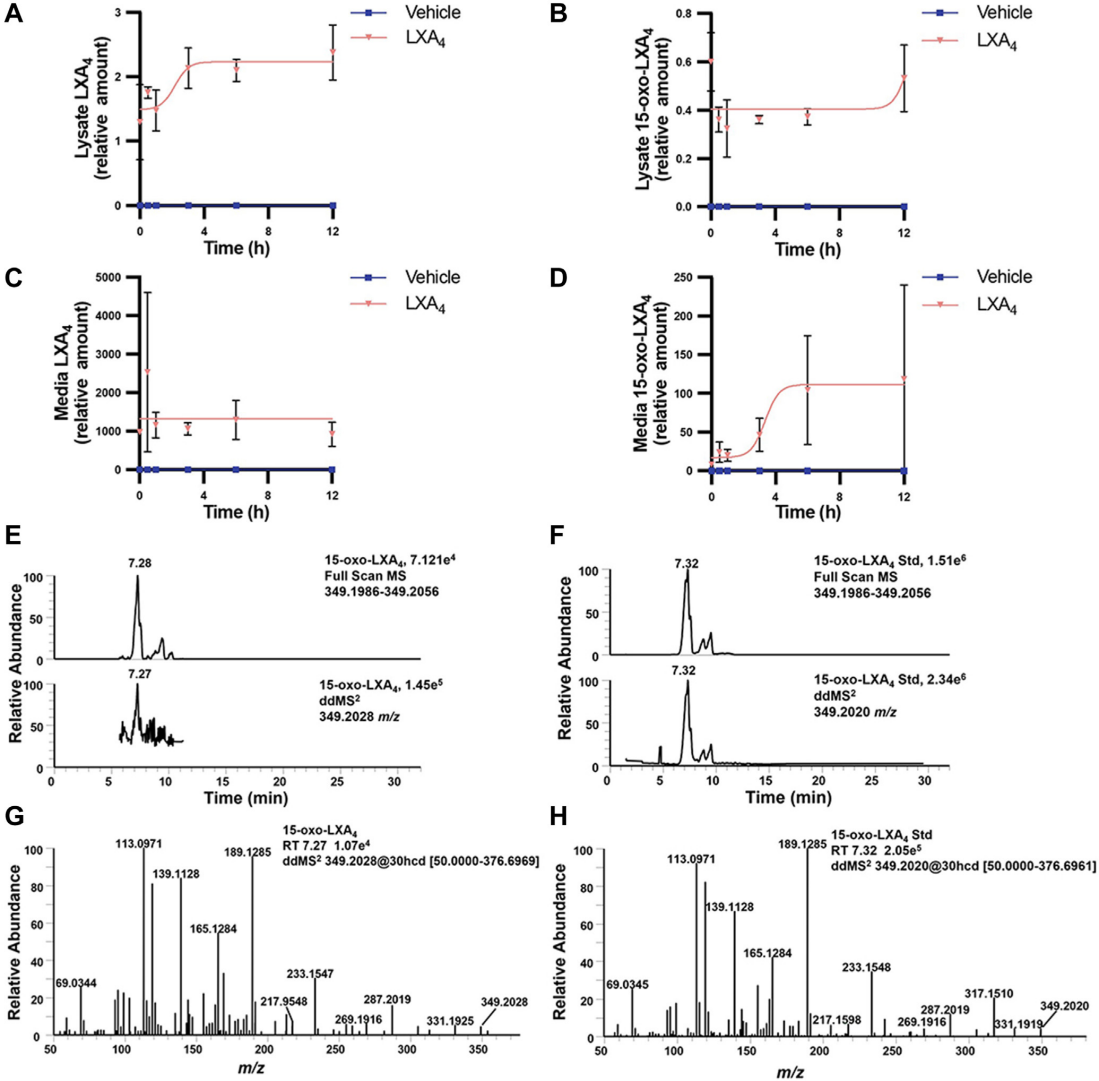
RAW264.7 macrophages metabolize exogenously-added LXA_4_ to 15-oxo-LXA_4_. RAW264.7 macrophages were treated with 25 μM LXA_4_ over a 12 h time course and (A) LXA_4_ and (B) 15-oxo-LXA_4_ were measured in cell lysate and (C) LXA_4_ and (D) 15-oxo-LXA_4_ were measured in cell media. E: The endogenously formed 15-oxo-LXA_4_ was retained at 7.28 min and detected in both full scan and ddMS^2^ with a *m/z* of 349.2028. F: 15-oxo-LXA_4_-Me was added to RAW264.7 cells for 1 h to create an ionizable standard for comparison of retention time and product ion MS^2^ and displayed matching *m/z* at 349.2020 at a retention time of 7.32 min. The product ion spectra of the (G) the endogenous 15-oxo-LXA_4_ analyte and (H) the 15-oxo-LXA_4_ standard have corresponding diagnostic fragment ions at *m/z* 331.1925, 233.1547, 189.1285, 165.1284, 139.1128, and 113.0971. Data is reported as Relative Amount, which is the peak area ratio of the analyte peak area divided by the peak area of LXA_4_-d_5_.

RAW264.7 macrophages were treated with 25 μM LXA_4_ over a 12 h period to investigate the formation of 15-oxo-LXA_4_. LXA_4_ was readily detected in the cell lysate (Fig. 4A), as was 15-oxo-LXA_4_ (Fig. 4B). Cell lysate levels of LXA_4_ increased within minutes and remained constant between 3 and 12 h, whereas intracellular levels of 15-oxo-LXA_4_ remained constant. Inversely, LXA_4_ levels remained constant in the media (Fig. 4C) and 15-oxo-LXA_4_ levels increased in the media over the first 6 h and remained constant between 6 and 12 h (Fig. 4D). 15-oxo-LXA_4_ eluted at a retention time of 7.28 min with *m/z* 349.2028 in both full scan and ddMS^2^ mode (Fig. 4E). To confirm the identity of 15-oxo-LXA_4_, cells were treated with synthetic 15-oxo-LXA_4_-Me that is rapidly de-esterified by methyl transferases (42). A peak having a retention time of 7.32 min was detected for the de-esterified 15-oxo-LXA_4_-Me product, with a parent *m/z* of 349.2020 for the M-H^+^ ion (Fig. 4F). The product ion spectra for 15-oxo-LXA_4_ and the de-esterified 15-oxo-LXA_4_-Me standard showed matching diagnostic ions at *m/z* 331.1919, 287.2019, 233.1548, 189.1285, 165.1284, 139.1128, 113.0971, and 69.0345 (Fig. 4G, H).

### Signaling actions of LXA_4_ and 15-oxo-LXA_4_-Me are FPR2-independent

**Fig. 5:**
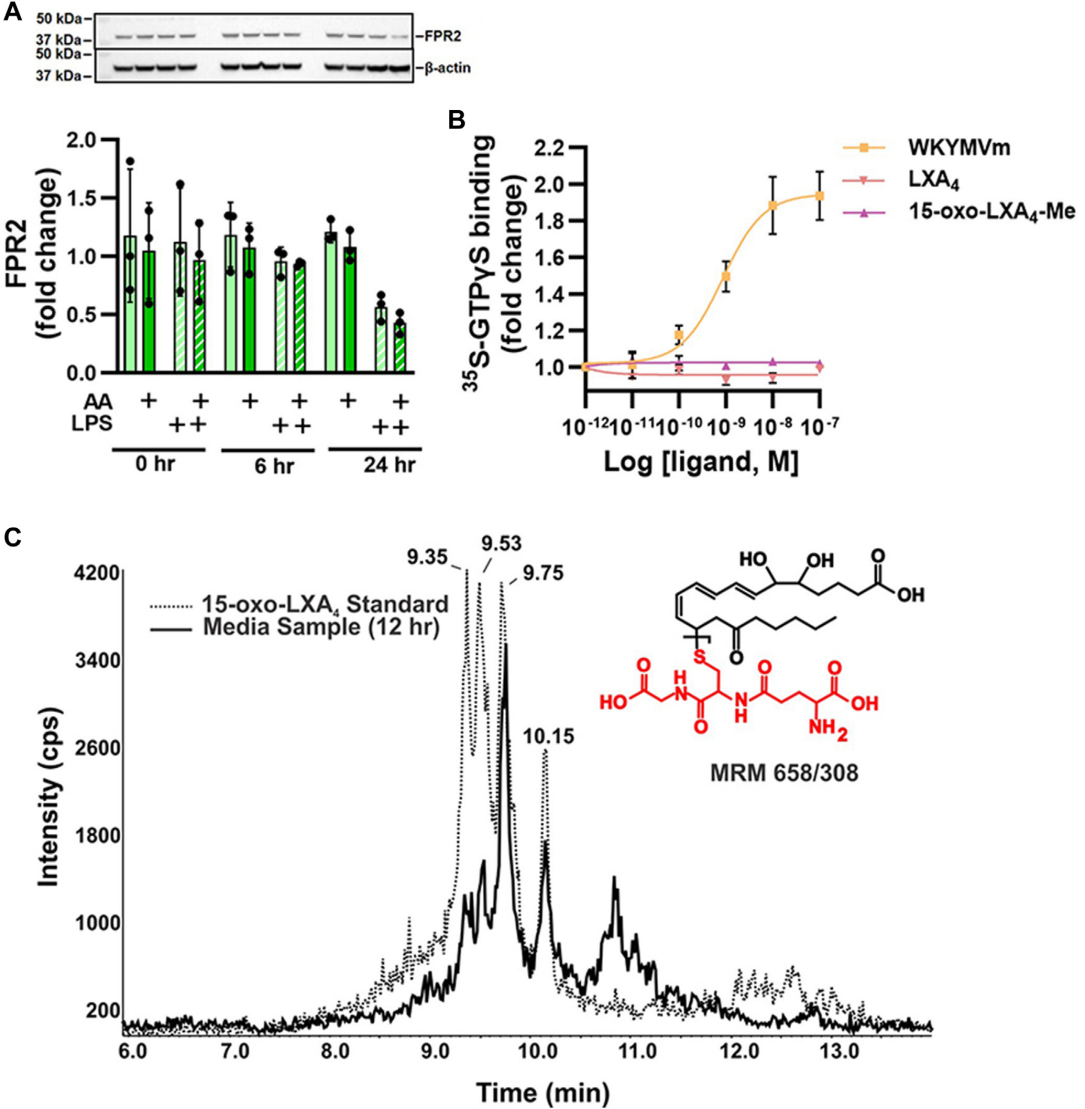
LXA_4_ and 15-oxo-LXA_4_-Me do not activate FPR2. A: FPR2 expression was measured by Western blot in RAW264.7 macrophages. B: Increasing concentrations of LXA_4_, 15-oxo-LXA_4_ and the FPR2 ligand WKYMVm were incubated with S-GTPγ and binding was determined. C: Chromatographic overlay of glutathionylated 15-oxo-LXA_4_ standard and 15-oxo-LXA_4_ glutathione adduct in RAW264.7 cell media at 12 h after treatment with 15-oxo-LXA_4_-Me. Immunohistochemical results are a representative blot that is consistent with data is from 3 independent experiments.

The FPR2 receptor is ligand-activated by the formylated peptide fMet-Leu-Phe and related fMet peptides, annexin-1, and amyloid-α and-β. FPR2 is the proposed receptor for LXA_4_ and other oxygenated unsaturated fatty acids (24, 25). Herein, the FPR2 receptor was expressed by RAW264.7 macrophages throughout the study period, with cell protein expression levels not affected by AA supplementation and LPS treatment (Fig. 5A). A sensitive in vitro GTPγS binding assay was performed to investigate the ligand-induced activation of FPR2 (47, 50). The assay measures as an indicator of FPR2 activation the binding of ^35^S-GTPγS to Gi, the cognate signaling partner of FPR2. The FPR2 peptide agonist, WKYMVm (51, 52) was used as a positive control as evidenced by its induction of significant FPR2 activation that promoted ^35^S-GTPγS binding to Gi. In contrast, LXA_4_ and 15-oxo-LXA_4_-Me did not display FPR2 receptor agonism at all concentrations tested (Fig. 5B).

To define whether 15-oxo-LXA_4_ is electrophilic and might signal via FPR2-independent mechanisms, 100 μM GSH and 10 μM 15-oxo-LXA_4_ (derived from 15-PGDH oxidation of LXA_4_) were incubated in 50 mM potassium phosphate buffer (pH = 8) for 1 h at 37 ˚C. Glutathionylated 15-oxo-LXA_4_ was monitored by LC-HRMS in positive ion mode using selected reaction monitoring at the *m/z* transition 658 → 308. This GSH adduct was found in cell supernatants of RAW264.7 cells treated with 25 μM 15-oxo-LXA_4_-Me. Multiple peaks are observed for glutathionylated 15-oxo-LXA_4,_ as the adduct can form through both a 1,4 or a 1,6 Michael addition; however, the 1,4 addition is more thermodynamically favorable (53, 54, 55). A representative chromatographic overlay is shown in Fig. 5C.

### 15-Oxo-LXA_4_-Me inhibits pro-inflammatory signaling

**Fig. 6:**
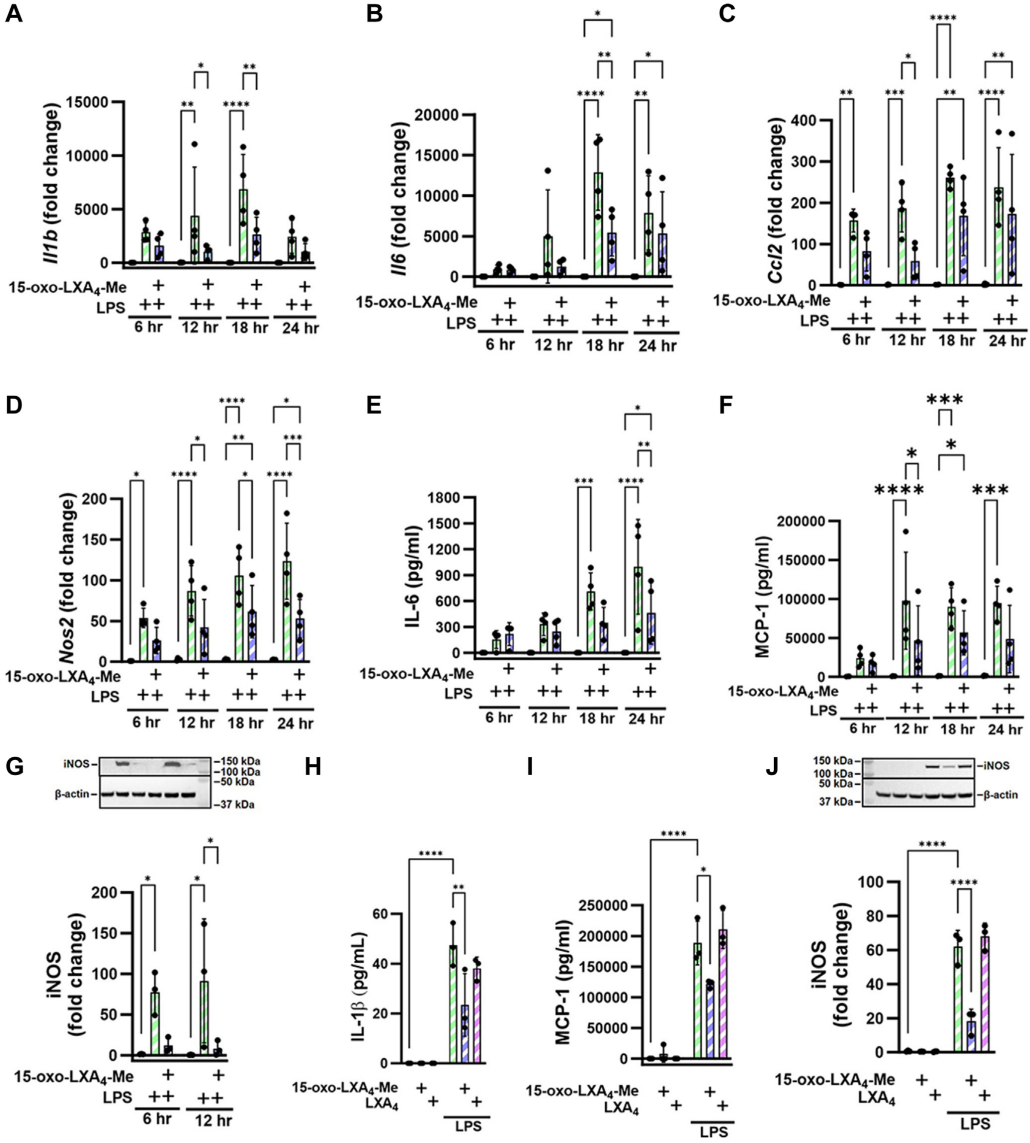
15-oxo-LXA_4_-Me inhibits pro-inflammatory cell signaling. Treatment of RAW264.7 macrophages with 15-oxo-LXA_4_-Me inhibits LPS-mediated upregulation of pro-inflammatory signaling across a 24 h period. (A) *Il1b*, (B) *Il6*, (C) *Ccl2*, (D) *Nos2* gene expression, (E) IL-6 protein, (F) MCP-1 protein and (G) iNOS protein expression. At 12 h 15-oxo-LXA_4_-Me and LXA_4_ inhibition of LPS-mediated upregulation of inflammatory pathways was compared for (H) IL-1β protein, (I) MCP-1 protein and (J) iNOS protein. Data is from three independent experiments ∗ *P* < 0.05, ∗∗*P* < 0.01, ∗∗∗*P* < 0.001, ∗∗∗∗*P* < 0.0001.

RAW264.7 macrophages were activated with LPS and the activation of NF-ĸB target gene expression was reflected by the increased expression of *Il1β*, *Il6*, *Ccl2*, and *Nos2* (as compared to vehicle controls (Fig. 6A–D). There was upregulation of IL-6 and MCP-1 protein expression measured by ELISA (Fig. 6E, F) and iNOS by Western blot analysis (Fig. 6G). The effects of 15-oxo-LXA_4_-Me on LPS-induced gene expression of pro-inflammatory mediators were variable and primarily occurred at the 12 and 18 h time points. LPS-induced *Il1β* expression was downregulated by 15-oxo-LXA_4_-Me at all measured times, becoming significant at 12–18 h (Fig. 6A). IL-6 mRNA expression and cytokine production was inhibited at most time points, with statistically significant decreases at 18–24 h after LPS plus 15-oxo-LXA_4_-Me treatment (Fig. 6B, E). MCP-1 mRNA expression and cytokine production were significantly inhibited by 15-oxo-LXA_4_-Me 12 h post-LPS administration (Fig. 6C, F). 15-oxo-LXA_4_-Me also significantly inhibited LPS-stimulated *Nos2* expression between 12 and 24 h (Fig. 6D). Compared with vehicle-treated cells, the maximum loss of viability due to LPS treatment was ∼20%, an effect that was mitigated by 15-oxo-LXA_4_-Me over the 24 h period. 15-oxo-LXA_4_-Me supplementation alone did not significantly impact cell viability (supplemental Fig. S1). The signaling actions of LXA_4_ were compared with 15-oxo-LXA_4_-Me in LPS-treated RAW264.7 macrophages at the 12 h time point. For the cytokines, IL-1β and MCP-1 (Fig. 6H, I) and iNOS protein expression (Fig. 6J), LXA_4_ did not significantly impact pro-inflammatory signaling mediator expression when compared to cells treated with 15-oxo-LXA_4_-Me.

### 15-Oxo-LXA_4_-Me induces anti-inflammatory and repair signaling

**Fig. 7:**
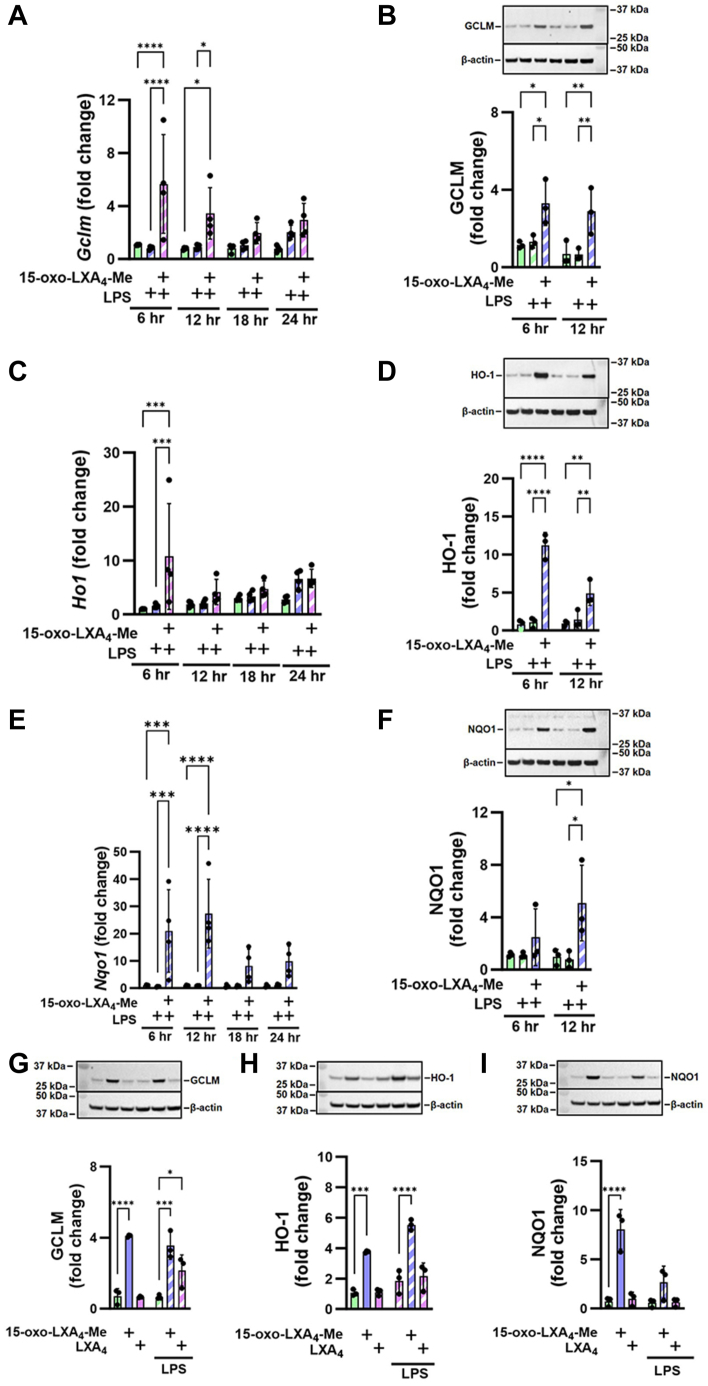
15-oxo-LXA_4_-Me activates Nrf2-regulated antioxidant and repair responses. Treatment of RAW264.7 macrophages with 15-oxo-LXA_4_-Me activates adaptive signaling responses upon LPS activation across a 24 h period. (A) *Gclm* (B) GCLM protein (C) *Ho1* (D) HO-1 protein (E) *Nqo1* (F) NQO1 protein. At 12 h, 15-oxo-LXA_4_-Me and LXA_4_ activation of the antioxidant response after LPS activation was compared for (G) GCLM protein (H) HO-1 protein and (I) NQO1 protein. Data is from three independent experiments ∗ *P* < 0.05, ∗∗*P* < 0.01, ∗∗∗*P* < 0.001, ∗∗∗∗*P* < 0.0001.

The activation of Nrf2-regulated gene and protein expression by 15-oxo-LXA_4_-Me was reflected by the increased expression of its targets including GCLM, (HO-1, and NQO1. 15-oxo-LXA_4_-Me increased both gene and protein expression of the Nrf2 regulated targets (Fig. 7A–F). *Gclm* and *Ho1* expression peaked at 6 h (Fig. 7A, C), while the increase in *Nqo1* expression at 6 h was sustained at 12 h (Fig. 7E). Western blotting of the Nrf2 target proteins further validated the activation of this electrophile-sensitive transcriptional regulatory mechanism by 15-oxo-LXA_4_-Me at 6 and 12 h for GCLM and HO-1 (Fig. 7B, D), and at 12 h for NQO1 (Fig. 7F). In contrast to robust 15-oxo-LXA_4_-Me responses, LXA_4_ had no significant impact on Nrf2-regulated adaptive signaling responses, both before and after RAW264.7 cell activation by LPS (Fig. 7G–I).

## Discussion

The formation of di- and tri-hydroxytetraene fatty acids in leukocytes has been studied by various groups in biochemical, cell culture, and *in vivo* systems since the 1980s (22, 23, 56). Although many cells express the enzymes required to synthesize these poly-hydroxylated fatty acid species, they are reported to be at very low or undetectable cellular and circulating concentrations. Also, reported shifts in these endogenous hydroxy-fatty acid concentrations are not definitively and temporally linked with FPR2 receptor occupancy and signaling responses that mediate the resolution of inflammation (6, 57, 58, 59, 60, 61). Notably, the initial reports of cellular LXA_4_ generation required neutrophil priming with the 15-lipoxygenase product 15-hydroperoxy-5,8,11,13-eicosatetraenoic acid (15-HPETE) to accomplish detectable lipoxin biosynthesis (13, 22). These and other issues motivated the present reexamination of the seminal observations of biological LXA_4_ generation by Serhan and Samuelsson in the 1980s. The current results provide additional perspective as to how hydroxylated fatty acids might signal, but only if present in sufficient concentrations. Herein, Western blot analysis affirmed the expression of 5-, 12/15-LO, and FLAP proteins that would be required for lipoxin biosynthesis in RAW264.7 macrophages (Fig. 2); however, LXA_4_ generation was undetectable upon LPS activation (supplemental Fig. S3A) and after supplementation of AA (Fig. 3 and supplemental Fig. S4). In contrast, under these conditions LPS activation induced both RAW264.7 macrophage and primary BMDM formation of prostaglandins and HETEs (Fig. 3A, supplemental Fig. S2–S5). Only upon supplementation of RAW264.7 macrophages with synthetic LXA_4_ was the 15-PGDH metabolite 15-oxo-LXA_4_ detected both intra- and extracellularly, eliminating the possibility that parent LXA_4_ was being formed and then rapidly metabolized (Fig. 4).

Endogenous LXA_4_ and other SPM concentrations are being re-evaluated by multiple labs due to a recent proposal that SPM levels can predict clinical and therapeutic outcomes, a precept that has been viewed to be based on flawed LC-MS/MS qualitative and quantitative approaches and that we view could lead to patient harm (36, 62). Investigators having expertise in MS-based lipidomics, including the present, are unable to replicate the biological detection of sufficient concentrations of trihydroxytetraene and other SPM that might induce or represent downstream signaling responses in vitro and in vivo (6, 9, 27, 63). This dilemma is magnified by a) the failure of the primary labs reporting biological SPM generation by LC-MS/MS to apply standard limit-of-detection or limit-of-quantitation criteria to SPM analyses, b) frequent incorrect and inadequate primary product and fragment ion identification, c) the use of misleading “representative” LC-MS chromatograms of SPM generation that are not directly linked with relevant primary data and d) a failure to provide primary analytical data upon request (36). These oversights and pitfalls in the assessment of chemically reactive redox metabolites exemplify “the triumph of hope over reality” (64). Specifically, many reports linking SPM generation with the resolution of inflammation come from the flawed characterization of background noise as the criterion for reporting the presence and concentrations of SPM in biological samples. These conundrums motivated the reassessment of past evidence for the presence of SPM in biological matrices and the inferred roles of LXA_4_ in the resolution of inflammation (62, 65, 66).

FPR2 receptor-independent reactions of 15-oxo-LXA_4_-Me are evidenced by the engagement of critical constituents of the redox-sensitive cysteine proteome (NF-ĸB and kelch-like ECH-associated protein-1/Nrf2-regulated gene expression) and the absence of FPR2 receptor agonism by both LXA_4_ and 15-oxo-LXA_4_ (Fig. 5, Fig. 6, Fig. 7). Current dogma holds that trihydroxytetraene oxidation to the 15-oxo metabolite by 15-PGDH inactivates FPR2 ligand activity and downstream signaling; This is not the case; however, as both LXA_4_ and 15-oxo-LXA_4_-Me displayed no ligand-induced activation of FPR2, as measured by GTPγS binding analysis. This contrasted with the robust activation of FPR2-GTPγS binding induced by WKYMVm, a synthetic peptide mimetic of N-formylated bacterial peptides, (Fig. 5B). While not excluding the remote possibility that both lipids may act on FPR2 to induce the activation of other FPR2 signaling partners such as β-arrestins, this would still require a sufficient concentration of LXA_4_ or its metabolite (4, 10, 25, 67, 68).

The pharmacokinetics of 15-oxo-LXA_4_, if present in sufficient concentrations, will distinctly differ from LXA_4_. Following oxidation of LXA_4_ to 15-oxo-LXA_4_, there will be an equilibrium between free and nucleophile-adducted 15-oxo-LXA_4_, as for other small molecule electrophiles. Over time, if LXA_4_ and 15-oxo-LXA_4_ generation is sufficient and continues, 15-oxo-LXA_4_-target protein adducts can accumulate and potentially induce downstream signaling responses from even low rates of LXA_4_ generation (69). This characteristic could explain how the administration of supra-physiological concentrations of synthetic LXA_4,_ as well as other SPMs and their prodrugs, will yield electrophilic α,β-unsaturated ketone containing metabolites that can contribute to the propagation of adaptive anti-inflammatory signaling in vivo. Present pharmacokinetic interpretations of LXA_4_ signaling rely on the weak GPCR ligand activity of LXA_4_, with Michaelis-Menten kinetics dictating downstream cellular responses. The present data indicates that the mechanisms accounting for the in vivo instigation of signaling responses by synthetic SPM-related species and their metabolites require more rigorous evaluation.

The generation and metabolism of HETEs is a well-established example of oxidation-induced functional switching of a hydroxy-fatty acid derivative to an electrophilic α,β-unsaturated ketone product. Free radical oxidizing species that are produced during digestion, basal metabolism, and inflammatory responses are capable of oxidizing unsaturated fatty acids to hydroperoxy derivatives, which are then reduced to hydroxy-fatty acids by peroxidases and further oxidized by dehydrogenases to α,β-unsaturated ketone products (70, 71). Enzymatically, 15-PGDH and other cellular dehydrogenases having broad substrate specificities generate α,β-unsaturated ketone products from HETEs. For example, the oxidation of 11-HETE and 15-HETE by 15-PGDH yields 11-oxo-eicosatetraenoic acid and 15-oxo-eicosatetraenoic acid, respectively (46, 72, 73). Both electrophilic metabolites promote adaptive anti-inflammatory responses by inhibiting NF-ĸB-dependent pro-inflammatory cytokine expression in murine and human macrophages (46, 73). Electrophilic metabolites of hydroxyl moieties of 20 carbon trihydroxypentadienes and 22-carbon trihydroxyhexadienes have been reported both in vitro and in vivo. For example, 8-oxo-resolvin D1 and 17-oxo-resolvin D1 are formed from resolvin D1 and 18-oxo-resolvin E1 from resolvin E1 (74, 75). These metabolites, originally viewed to have lost ligand activity for their cognate GPR32, GPR18, and/or chemerin receptor 23 receptors, have yet to be characterized in the context of the expected broad range of downstream signaling actions of their corresponding electrophilic α,β-unsaturated ketone-containing metabolites (19, 74).

In summary, the electrophilic metabolite of LXA_4_, 15-oxo-LXA_4_, activated Nrf2-regulated antioxidant and tissue repair responses and inhibited LPS-induced, NF-ĸB-regulated pro-inflammatory cytokine expression in RAW264.7 macrophages. Many of the SPM properties attributed to LXA_4_ and other mono-, di- and trihydroxy fatty acids overlap with those reported for electrophilic fatty acids and other endogenous small molecule electrophiles. The present results indicate that the LXA_4_ metabolite 15-oxo-LXA_4_ and other more abundant electrophilic omega-3 fatty acid mono-hydroxy derivatives can exert electrophile-responsive, FPR2 receptor-independent effects after further oxidation. For this property to be of biological significance in the context of endogenously generated SPM; however, LXA_4_ and related SPM must be generated at sufficient concentrations. This latter dilemma affirms the ongoing critical evaluation of previously reported primary data and recalibration of SPM pharmacodynamics. The extensive literature describing the multi-target signaling actions of lipid electrophiles indicates that most products of non-enzymatic and enzymatically-catalyzed fatty acid oxidation reactions can induce antioxidant and anti-inflammatory responses upon alkylation of highly conserved nucleophilic amino acids of enzymes, transcriptional regulatory proteins and ion channels (70, 76, 77). In aggregate, the present data motivates a reexamination of the reported endogenous concentrations of LXA_4_ and other SPM-related oxylipins in the context of primary mass spectral evidence and a reexamination of the gene expression responses and the temporal differentiation of GPCR-dependent and GPCR-independent responses to unsaturated fatty acid hydroxyl and oxo fatty acid metabolites; all of which must be scrutinized with scientific rigor.

## Data availability

Data are in the manuscript and raw MS data files are available upon request (Stacy L. Gelhaus, gstacy@pitt.edu)

## Supplemental data

This article contains supplemental data.

## Conflict of interest

The authors declare the following financial interests/personal relationships which may be considered as potential competing interests: BAF acknowledges an interest in Creegh Pharmaceuticals.
