# Supplementary material for: Lipoxin A4 yields an electrophilic 15-oxo metabolite that mediates FPR2 receptor-independent anti-inflammatory signaling
Source: J Lipid Res. 2024 Nov 19;66(1):100705. doi: 10.1016/j.jlr.2024.100705 (PMC11729656; doi:10.1016/j.jlr.2024.100705)
Supplement: Supplemental data [file mmc1.pdf]

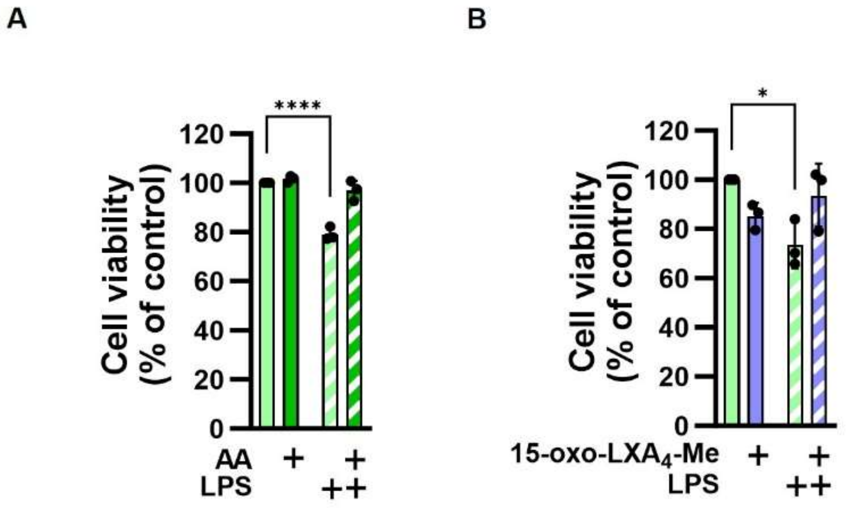

Supplementary Figure 1. MTT cell viability. (A) RAW264.7 macrophages were supplemented with arachidonic acid (AA) alone or supplemented with AA and activated with LPS. Cell viability was measured at 24 h. (B) RAW264.7 macrophages were treated with 15-oxo-LXA_4 alone or treated with 15-oxo-LXA_4 and activated with LPS. Cell viability was measured after 24 h. * $p < 0.05$ and **** $p < 0.0001$.

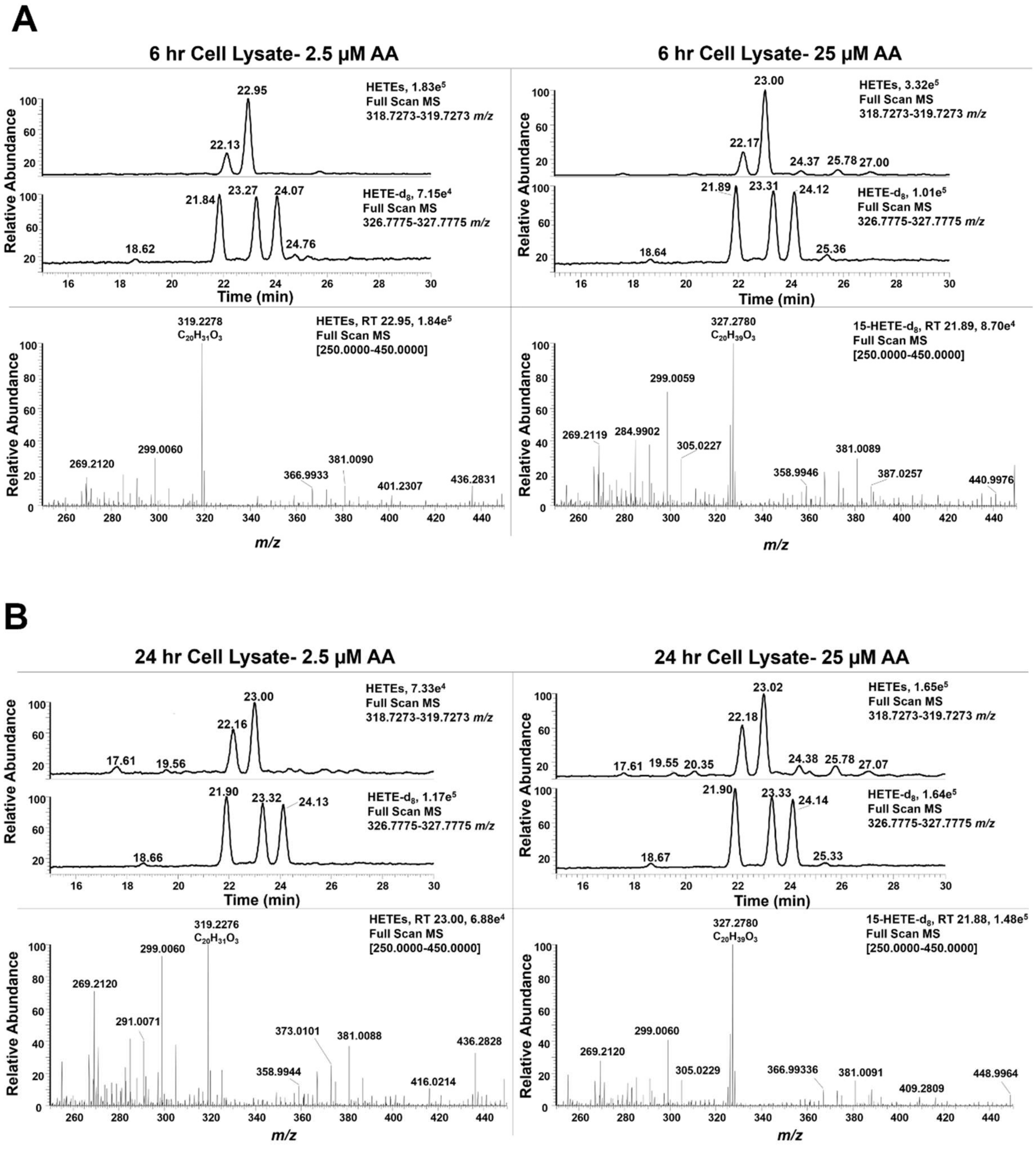

Supplementary Figure 2. RAW264.7 macrophage produce hydroxyeicosatrienoic acids. HETEs are present in both cell lysate at 6 h (A) and 24 h (B) with 10 ng/mL LPS activation and 2.5 µM or 25 µM arachidonic acid supplementation. HETE internal standards have an *m/z* of 327.2775 and the following retention times 15-HETE-d_8 (21.9 min), 12-HETE-d_8 (23.3 min), and 5-HETE-d_8 (24.1 min). Peaks with *m/z* 319.2273 for endogenous HETE species are detected at 22.1, 23.0, and 24.3 min, likely corresponding to 15-HETE, 11-HETE and 5-HETE, respectively.

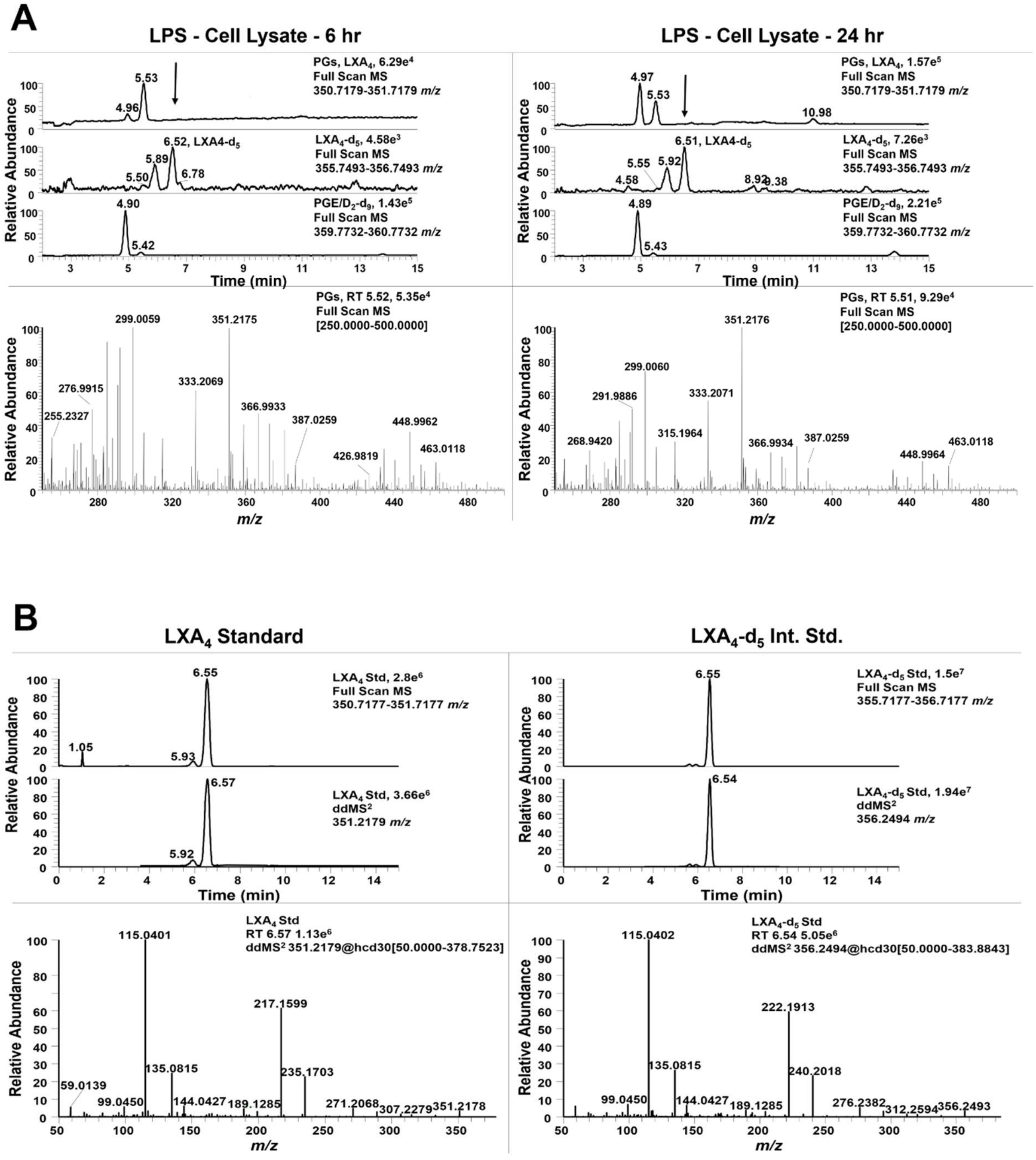

Supplementary Figure 3. LPS activation of RAW264.7 macrophage does not produce LXA$_4$. (A) RAW264.7 macrophage were activated with 10 ng/mL LPS for 6 and 24 h. Activation did not result in the formation of LXA$_4$ at either time point. (B) LXA$_4$ standard chromatogram in Full Scan, ddMS^2 at *m/z* 351.2179 and Full MS^2 product ion spectra. LXA$_4$-d$_5$ internal standard chromatogram in Full Scan and ddMS^2 at *m/z* 356.2494 and Full MS^2 product ion spectra. Product ion spectra for LXA$_4$ and LXA$_4$-d$_5$ show corresponding diagnostic ions at *m/z* 351.2178/356.2493, 307.2279/312.2594, 271.2068/276.2382, 235.1703/240.2018, 217.1599/22.1913, and unlabeled ions at 135.0815 and 115.0401.

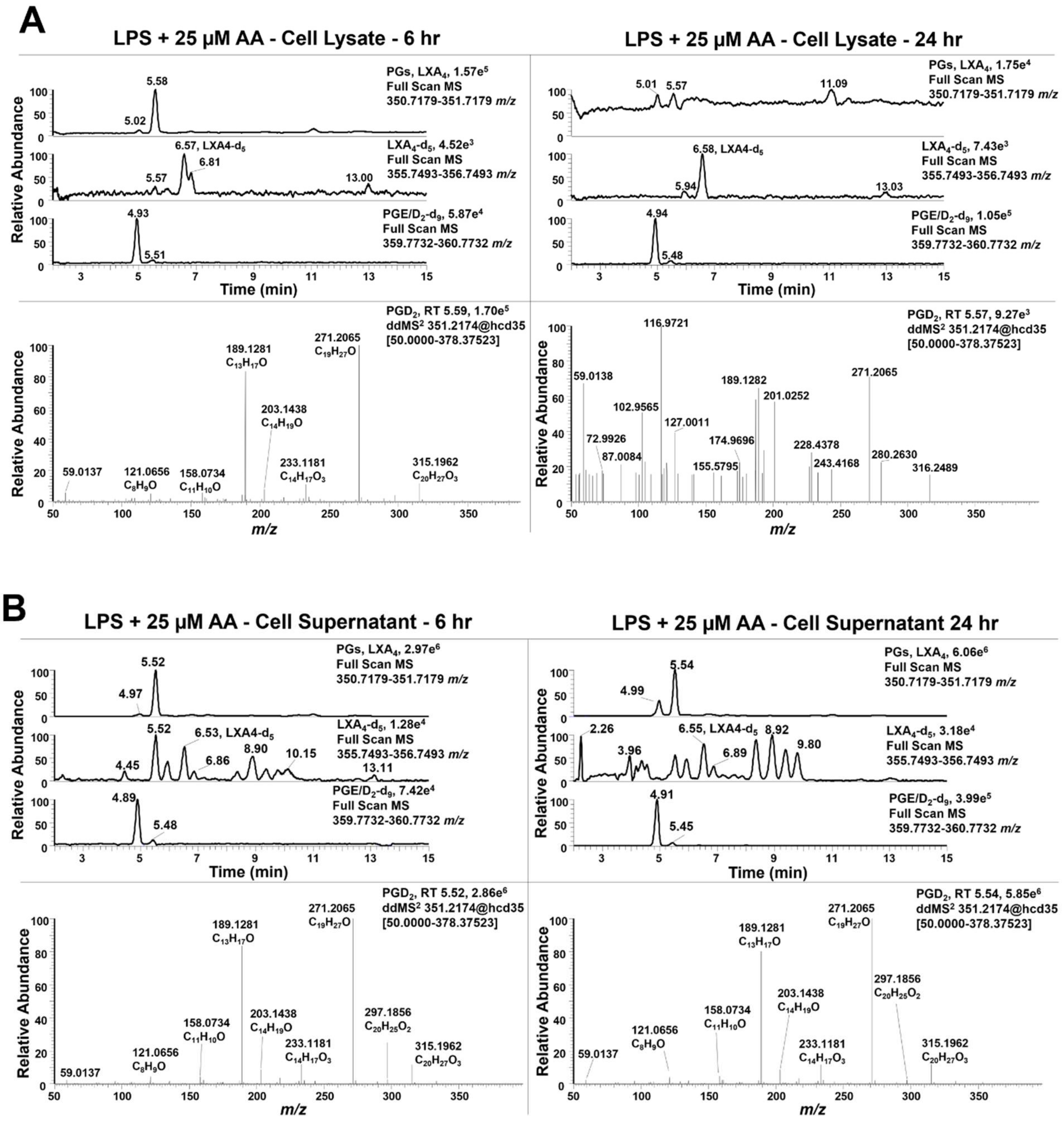

Supplementary Figure 4. LPS activation and 25 µM arachidonic acid supplementation of RAW264.7 macrophage does not produce LXA_4. RAW264.7 macrophage were activated with 10 ng/mL LPS and supplemented with 25 µM AA for 6 and 24 h. LXA_4 is not detected in (A) cell lysate or (B) cell supernatant. Prostaglandins *m/z* 351.2174, PGE_2 (~4.9 min) and PGD_2 (~5.5 min) are found at both time points in both cell lysate and cell supernatant. The product ion spectra for PGD_2 at ~5.5 min displays diagnostic product ions at *m/z* 315.1962, 217.2065, and 189.1281 at both 6 and 24 h.

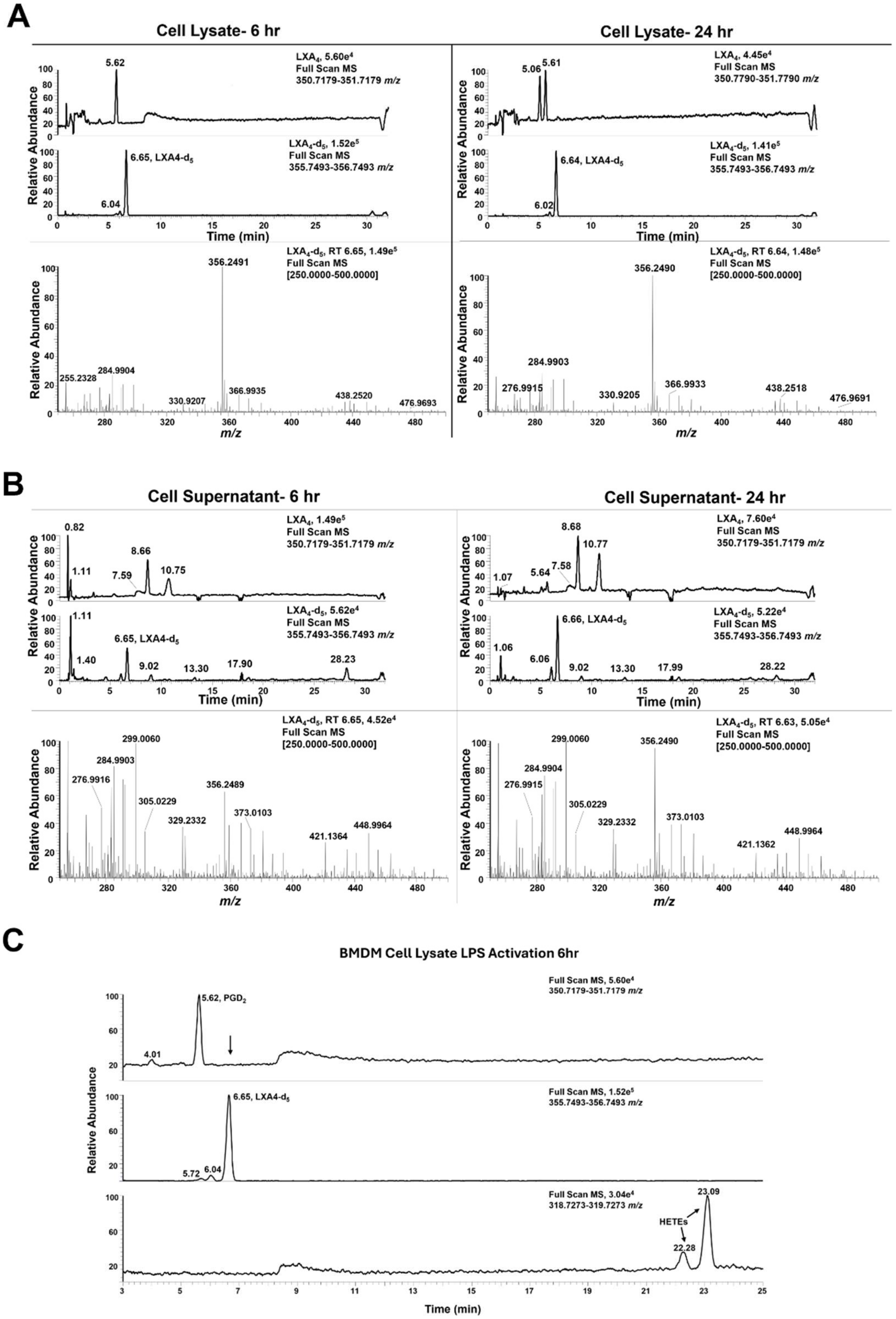

Supplementary Figure 5. Bone marrow derived macrophage do not produce LXA$_4$. Bone marrow derived macrophage taken from naïve C57BL6/J mice were activated with 10 ng/mL LPS. LXA$_4$ was not detected in the cell lysate (A) or the cell supernatant (B) at 6 and 24 h. Prostaglandins and HETEs were detected in cell lysate (A and C).
